# Nanocrystalline Hydroxyapatite Loaded with Resveratrol in Colloidal Suspension Improves Viability, Metabolic Activity and Mitochondrial Potential in Human Adipose-Derived Mesenchymal Stromal Stem Cells (hASCs)

**DOI:** 10.3390/polym11010092

**Published:** 2019-01-08

**Authors:** Krzysztof Marycz, Agnieszka Smieszek, Justyna Trynda, Paulina Sobierajska, Sara Targonska, Lukasz Grosman, Rafal J. Wiglusz

**Affiliations:** 1 Department of Experimental Biology, Faculty of Biology and Animal Science, Wroclaw University of Environmental and Life Sciences, ul. Norwida 27B, 50-375 Wroclaw, Poland; agnieszka.smieszek@upwr.edu.pl (A.S.); justyna.trynda@upwr.edu.pl (J.T.); 2 Faculty of Veterinary Medicine, Equine Clinic-Equine Surgery, Justus-Liebig-University, 35392 Giessen, Germany; 3 Institute of Low Temperature and Structure Research, Polish Academy of Sciences, Okolna 2, 50-422 Wroclaw, Poland; p.sobierajska@intibs.pl (P.S.); s.targonska@intibs.pl (S.T.); l.grosman@hasco-lek.pl (L.G.); r.wiglusz@intibs.pl (R.J.W.); 4 Centre for Advanced Materials and Smart Structures, Polish Academy of Sciences, Okolna 2, 50-950 Wroclaw, Poland

**Keywords:** hydrogels, 3,6-anhydro-α-l-galacto-β-d-galactan, resveratrol, nanocomposites

## Abstract

In response to the demand for new multifunctional materials characterized by high biocompatibility, hydrogel (HG) nanocomposites as a platform for bioactive compound delivery have been developed and fabricated. A specific crosslinking/copolymerization chemistry was used to construct hydrogels with a controlled network organization. The hydrogels were prepared using 3,6-anhydro-α-l-galacto-β-d-galactan (galactose hydrogel) together with resveratrol (trans-3,5,4′-trihydroxystilbene) and calcium hydroxyapatite nanoparticles. The resveratrol was introduced in three different concentrations of 0.1, 0.5, and 1 mM. Nanosized calcium hydroxyapatite was synthesized by a microwave-assisted hydrothermal technique, annealed at 500 °C for 3 h, and introduced at a concentration 10% (*m*/*v*). The morphology and structural properties of Ca_10_(PO_4_)_6_(OH)_2_ and its composite were determined by using XRPD (X-ray powder diffraction) techniques, as well as the absorption and IR (infrared) spectroscopy. The average nanoparticle size was 35 nm. The water affinity, morphology, organic compound release profile, and cytocompatibility of the obtained materials were studied in detail. The designed hydrogels were shown to be materials of biological relevance and of great pharmacological potential as carriers for bioactive compound delivery. Their cytocompatibility was tested using a model of human multipotent stromal cells isolated from adipose tissue (hASCs). The biomaterials increased the proliferative activity and viability of hASCs, as well as reduced markers of oxidative stress. In light of the obtained results, it has been thought that the designed materials meet the requirements of the tissue engineering triad, and may find application in regenerative medicine, especially for personalized therapies.

## 1. Introduction

Multifunctional hybrid materials, i.e., those that are organic/inorganic, are able to respond, reversibly, to more than one external stimulus. Such enhanced materials exhibit an advantage over those showing a single-responsive behavior. For clinical treatment, single-responsive materials leave a great deal to be desired, in the case of an effective therapy for patients with more than one symptom. In order to resolve these drawbacks, many researches have proposed new strategies aimed to obtain multifunctional materials that could be safely adjusted to ensure successful treatment [1]. Targeted drug delivery systems promise improved therapy due to the direct transportation of the drug into a certain tissue. Controlling drug release using a polymer carrier with a known release profile allows the diffusion mechanism to be analytically adjusted, providing safer therapies [2]. Nowadays, regenerative medicine and tissue engineering have become the fastest developing field of medicine. Taking into account the rapid aging of populations worldwide, especially in developed countries, advanced therapies are required to improve the regenerative processes of damaged tissue and/or organs [3,4]. The combination of various types of materials, together with progenitor cells including mesenchymal multipotent stromal cells, hold great promise in the treatment of many types of diseases [5]. Regenerative medicine based on the application of cell therapies and tissue engineering allows for restoring the loss of function of damaged tissue through improvement of cell proliferation and viability, as well as formation of a proper extracellular matrix [3,6,7]. Moreover, the desired materials or scaffolds for personalized medicine, except for their “bridging function”, should also deliver bioactive agents affecting the cell cytophysiology and enhance the formation of functional tissue [8,9]. Therefore, the triad, i.e., progenitor cells combined with multifunctional polymer as the scaffold and bioactive compounds, have been recognized as functional components of a regenerative therapy [10].

In the case of materials designated for regenerative medicine, hydrogels have received attention because of their innate structural and compositional similarities to the extracellular matrix, and their extensive framework for cellular proliferation and survival [11,12,13]. Many different hydrogels with various chemical and physical properties have been developed over the last several decades from a wide variety of chemical building blocks [14,15,16]. Knowledge regarding the hydrogels’ chemistry helps to improve the scaffold properties, such as molecular response, structural integrity, biodegradability, biocompatibility, and solute transport. Thus, the hydrogels are carefully engineered to meet the cytophysiological demands of progenitor cells, such as attachment, proliferation, and differentiation [12]. Special attention is paid to 3,6-anhydro-α-l-galacto-β-d-galactan because the physical building blocks utilized in tissue engineering should be safe and natural [17].

Therapies based on the application of progenitor cells derived from an adult organism, such as multipotent mesenchymal stromal cells (MSCs, also known as adult stem cells) represent one of the most important and powerful tools in regenerative medicine. The MSCs provide a realistic source of progenitor cells, and could be used for the treatment of age-related musculoskeletal and joint diseases, diabetes, and neurodegenerative diseases. Multipotent stromal cells originate from various tissues of mesenchymal origin, e.g., bone marrow and adipose tissue [6,18]. At the moment, ASCs are extensively used, especially in veterinary regenerative medicine, although human clinical trials are conducted as well [19]. The regenerative properties of ASCs are explained by their paracrine activity, by secretion of combinations of trophic factors modulating and improving intercellular signaling [20]. However, our previous report showed that ASCs lose their regenerative properties with patient age [21]. Hence, this seems to be serious limitation for their clinical application in the case of the personalized medicine [22]. Our and the other studies have been shown that the main factor limiting trophic activity of the ASCs is oxidative stress (OS) together with reduced superoxide dismutase (SOD) activity. As a consequence, impaired mitochondrial activity limits the ASCs’ regenerative properties [23,24]. Therefore, a reasonable strategy can be related to new factors that could improve mitochondrial activity through reducing oxidative stress. 

Resveratrol (RES, *trans*-3,5,4′-trihydroxystilbene), a plant-derived polyphenol, is a non-flavonoid antioxidant with various anti-aging and immunomodulatory effects. It has been shown that RES improves MSCs’ proliferative and differentiation potential, even after culturing on chitosan–gelatin scaffolds [25]. It has been noted that the RES promotes the expression of osteolineage genes, while suppressing adipolineage genes [26]. Our previous research showed that incorporation of RES into polyurethane–polylactide-based material decreases senescence and oxidative stress of human ASCs [23]. In the current study, nanocrystalline hydroxyapatite (nHAp) has been used with the general chemical formula Ca_10_(PO_4_)_6_(OH)_2_ as a carrier for RES. Nanosized HAp has been considered to be the most biocompatible material for bone regeneration [27,28]. Natural calcium hydroxyapatite obtained from patient bone (auto/allo/xenograft), in contrast to the synthetic one, exhibits the best biocompatible properties. As has been shown, synthetic calcium apatite in nanometric size is characterized by high cytocompatibility and osteoconductive effect, as well as demonstrating bioactive behavior by integrating with living tissue [29]. It is well known that calcium apatite supports osteoblast adhesion and proliferation [30]. Moreover, the nHAp can be modified with an active antibacterial agent (e.g., tetracycline) promoting simultaneously viability and proliferative activity of MSCs [31]. Furthermore, the nHAp effectively promotes the osteogenic differentiation potential of the MSCs, and could be used as an efficient tool for drug and/or a bioactive agent delivery system [32,33]. It is worth noting that nHAp doped with lanthanide ions also exhibits luminescent ability, which makes them an excellent candidate for bioimaging [34]. Besides, used of calcium apatite as biomaterial is limited due to its poor mechanical properties, therefore, it is primarily used as a filler or a coating on orthopedic and dental implants [29,35,36]. In this study, a new strategy has been proposed for the deposition of calcium hydroxyapatite into biodegradable hydrogels. This modification has led to the mechanical improvement of the material, preserving its biocompatibility and providing possible controlled release at the target site of action. It has been shown that the resveratrol-functionalized nanohydroxyapatite in three different dosages with human adipose-derived mesenchymal stem cells (hASCs) could be a potential biomaterial for bone tissue regeneration/reconstruction. The cytocompatibility of obtained materials was evaluated in vitro using human ASCs. Moreover, it has been shown that particular doses of RES can promote the viability of the ASCs, proliferative and metabolic activity, as well as mitochondrial potential, reducing oxidative stress. In this way, nHAp modified with RES could be become a future promising material for regenerative medicine.

## 2. Materials and Methods

### 2.1. Synthesis of Hydroxyapatite Nanopowders

The nanocrystalline powders of hydroxyapatite were prepared by the microwave-stimulated hydrothermal method. Analytical grade Ca(NO_3_)_2_∙4H_2_O (99% Acros Organics, Gdansk, Polska), NH_4_H_2_PO_4_ (99.995% Alfa Aesar, Haverhill, MA, USA), and NH_4_OH (99% Avantor Performance Materials Poland S.A, Gliwice, Poland) were used for pH adjustment. At first, a stoichiometric amount of calcium nitrate was dissolved in deionized water. A suitable amount of ammonium dihydrogen phosphate was added to the mixture, leading to fast precipitation of the byproduct. The pH of the dispersion was modulated to 9–10 by addition of ammonia, transferred into the Teflon vessel, and set in the microwave reactor (ERTEC MV 02-02, Ertec-Poland, Wroclaw, Poland). After 90 min of microwave-stimulated hydrothermal processing at 280 °C and under autogenous pressure of 60 atm, nanocrystalline powders were obtained. Finally, the product was washed with deionized water, dried at 70 °C for 24 h, and heat-treated at 500 °C for 3 h.

### 2.2. Preparation of 3,6-Anhydro-α-l-galacto-β-d-galactan Samples Filled with Hydroxyapatite and Resveratrol

**Figure 1:**
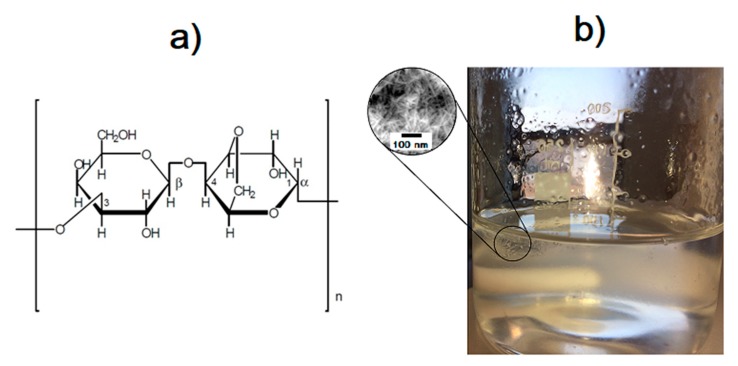
Chemical structure of the polysaccharide unit of 3,6-anhydro-α-l-galacto-β-d-galactan molecules (**a**) and the prepared hydrogel (**b**).

In order to produce the 3,6-anhydro-α-l-galacto-β-d-galactan hydrogel matrix, pure 3,6-anhydro-α-l-galacto-β-d-galactan was first dissolved in deionized water and heated to around 120 °C. Nanohydroxyapatite was added to a concentration of 10% (*m*/*v*) and resveratrol (*trans*-3,5,4′-trihydroxystilbene) to concentrations of 0.1, 0.5, and 1 mM. The components were added in sequence and mixed constantly for total homogenization. Finally, glycerine was added and the obtained colloid was cooled to room temperature (please see Figure 1). The product was left on a petri dish to permanently gel, then was subjected to lyophilization process to obtain a porous gel form.

### 2.3. Characterization

Powder X-ray diffraction of samples have been measured by using a PANalytical X’Pert Pro X-ray diffractometer (Malvern Panalytical Ltd., Royston, UK) in 2θ range of 5–80° equipped with Ni-filtered Cu Kα1 radiation (Kα1 = 1.54060 Å, *U* = 40 kV, *I* = 30 mA). The measurement result was collated with the standards obtained from the Inorganic Crystal Structure Database (ICSD) and Powder Diffraction File (PDF). 

The absorption spectra were analyzed by an Agilent Cary 5000 UV–Vis–NIR spectrophotometer (Agilent Technologies, Santa Clara, CA, USA) employing spectral bandwidth of 0.1 nm in the visible range and 0.2 nm in the near-infrared. The spectra have been recorded between 200 and 800 nm wavelengths. The attenuated total reflectance ATR-FTIR measurements were performed using FTIR spectrometer Nicolet iS50 from ThermoFisher Scientific (Waltham, MA, USA) using a diamond crystal and DLaTGS-KBr detector. The measurements were performed in the range 4000–600 cm^−1^, and the spectral resolution was set at 4 cm^−1^ with 32 scans. 

In order to observe the surface morphology of samples, a scanning electron microscope equipped with energy dispersive spectroscopy FEI Nova NanoSEM 230 (FEI Company, Hillsboro, OR, USA) equipped with an energy dispersive spectrometer (EDAX PegasusXM4) with an acceleration voltage of the range 3.0–15.0 kV and spot 2.5–3.0 has been used. Before observation, all of the samples have been sprayed uniformly with a layer of graphite.

In order to check the particle size and morphology, HRTEM was performed, using a Philips CM-20 Super Twin microscope (FEI Company, Hillsboro, OR, USA), operated at 200 kV with a resolution of 0.24 nm. The samples were deposited on a Cu grid with a carbon film from a suspension in ethanol. Afterwards, a droplet of suspension was deposited on a copper microscope grid covered with perforated carbon. The primary size of particles was evaluated using volume weighted formula:

$$d_{dv}=\frac{\sum n_{i}d_{i}^{4}}{\sum n_{i}d_{i}^{3}},$$

where *d_av_* is the average particle size, *n* the number of particles, and *d* represents particle diameter.

### 2.4. Determination of Resveratrol Release from 3,6-Anhydro-α-l-galacto-β-d-galactan Hydrogel Filled with Resveratrol and Hydroxyapatite

#### 2.4.1. Dissolution Method

A flow-through cell dissolution apparatus, SOTAX AG CE-7 SMART (Sontax AG, Aesch, Switzerland), with closed loop, was used in experiments. In all experiments, laminar flow was used. The degassed dissolution medium, phosphate buffer pH = 6.8 at 37.0 ± 0.5 °C, was pumped at a flow rate of 8 mL/min through small tablet cells (12 mm i.d.). For each interval, samples were collected in HPLC vials at the following times: 1, 3, 5, 8, 24, and 48 h. The concentration of each sample was determined using HPLC method.

#### 2.4.2. HPLC Method

Resveratrol was analyzed using the HPLC method (Agilent 1260 Infinity system, Agilent Technologies Inc., Santa Clara, CA, USA) using reverse phase column C18 (5 μm particle size, 250 mm × 4.6 mm, Phenomenex, Torrance, CA, USA) at 35 °C. The mobile phase was a mixture of phosphate buffer pH = 6.8, acetonitrile, and methanol 30:7:63 (*v*/*v*/*v*). The autosampler temperature was set at 4 °C. The injection volume was 100 μL, flow rate 1 mL/min, and run time 10 min. The amount of resveratrol dissolved was determined at 306 nm.

### 2.5. Human Adipose-Derived Multipotent Stromal Cells (hASCs)

The method of human adipose-derived multipotent stromal cells (hASCs) isolation was presented by Smieszek et al., in detail [37]. Human subcutaneous adipose tissue samples were collected from both male and female subjects, age range 20–33 (*n* = 8, mean age 24 ± 1.5 years). All donors had given their written consciously consent prior to the procedure. The protocol was approved by the Local Bioethics Committee of Wroclaw Medical School (registry number KB-177/2014, Wroclaw, Poland). Primary cultures of hASCs were propagated following our previous well-established protocol [33,38]. Immunophenotyping of hASCs was performed after third passage (p3). 

### 2.6. The Influence of Hydrogels Functionalized with nHAp and Resveratrol on Cultures of hASCs

Cytocompatibility of hydrogels was tested using hASCs at p5. The hASCs at 2 × 10^4^ cells per well were suspended in a 0.5 mL of complete growth medium, and seeded on 24-well plates covered with biomaterials. The morphology, metabolism, proliferative activity, and cytotoxicity were assessed. All experiments were performed in 10 independent repetitions. Materials used for accomplishment experiments were obtained from Sigma-Aldrich (Poznan, Poland).

#### 2.6.1. The Analysis of Metabolic and Proliferative Activity of Human ASCs. The Alamar Blue Test and Bromodeoxyuridine hASC Proliferation Assay

Human ASCs were assessed using a resazurin-based assay (Alamar Blue, Sigma-Aldrich, Poznan, Poland) for the following incubation times: 24, 48, and 72 h. The procedure was performed according to a well-established protocol that was described previously [39]. The metabolic activity was resented as ∆∆A value, which refers to the difference between the absorbance read at 600  and 690 nm, including calibration with a blank sample [40]. Additionally, the population doubling time was determined using the method presented in Marycz et al. [41], and an online calculator [42].

The evaluation of cell proliferation was carried out by a BrdU Cell Proliferation ELISA Kit ELISA test (Abcam, Symbios, Straszyn, Polska) according to the manufacturer’s protocol. After 48 h, hASCs were collected from biomaterials using trypsin solution (TrypLE™ Express, Thermo Fisher Scientific, Warszawa, Poland), and transferred to six wells of a 96-well plate. In the BrdU cell proliferation assay, BrdU, a pyrimidine analogue is incorporated into replicating DNA instead of thymidine, and then immunodetection of BrdU using specific monoclonal antibodies allows labeling of the cells in the S phase of the cell cycle. The optical density of the samples was determined at 370 nm. Spectrophotometric measurements were required both for resazurin, as well as the BrdU-based assay, and were performed using a Spectra MaxPlus Reader (Molecular Devices, Syngen, Wroclaw, Poland).

#### 2.6.2. Cytotoxicity of Hydrogels Functionalized with nHAp and Resveratrol toward hASCs

The influence of biomaterial on cell viability was assessed after 72 h of propagation. Apoptosis detection was performed using Muse^®^ Annexin V and Dead Cell Assay Kit (Merck, Warszawa, Poland) following the manufacturer’s protocol. The assay allowed differentiation of apoptotic cells (early and late apoptosis) from necrotic cells. For the analysis, cells were detached from biomaterial and polystyrene surfaces using trypsin solution (TrypLE™ Express, Thermo Fisher Scientific, Warszawa, Poland), centrifuged at 300× *g* for 4 min, and then stained with dye. Each experiment was performed in three independent repetitions. The analysis was performed on the Muse™ Cell Analyzer (Merck, Warszawa, Poland).

#### 2.6.3. The Analysis of Biomaterials Cytotoxicity Based on Mitochondrial Membrane Potential of hASCs

An epifluorescence microscope (EpiFM; Olympus Fluoview FV1200) was used for visualizing the mitochondria of hASCs cultured on the investigated biomaterials. Mitochondria were stained with MitoRed dye (1:1000). The protocol included incubation of cultures with MitoRed in 37 °C for 30 min in a CO_2_ incubator, fixation with 4% paraformaldehyde, staining with ATTO 488-labeled phalloidin (1:800), and application onto a microscope slide with ProLong™ Mountant with DAPI (Thermo Fisher Scientific, Warszawa, Poland). Data were collected using an ORCA-Flash 4.0 V3 Digital CMOS camera. The Muse MitoPotential kit (Merck, Warszawa, Poland) was used for estimation of the mitochondrial membrane potential. Human ASCs were stained according to the manufacturer’s protocol. For the quantitative measurements of reactive oxygen species (ROS), cell pellets were stained with The Muse^®^ Oxidative Stress Kit (Merck Millipore, Burlington, VT, USA).

#### 2.6.4. The Analysis of Human ASCs Morphology Using Scanning Electron Microscope (SEM)

The morphology of the human ASC was evaluated 72 h of the experiment using SEM. For this purpose, the human ASC cultures propagated with tested biomaterials were fixed with 4% paraformaldehyde (PFA). Then, cultures were rinsed with distilled water, and dehydrated in a graded ethanol series (concentrations from 50% to 100%, every 5 min). Samples were then sprinkled with gold particles using a 300 s program (Edwards, Scancoat six, HHV Ltd., Crawley, UK), transferred to the microscope chamber, and observed using an SE1 detector at 10 kV of filament tension (SEM, Evo LS 15, Zeiss, Oberkochen, Germany). Microphotographs were captured at 2500× magnification.

#### 2.6.5. Real-Time Reverse Transcription Polymerase Chain Reaction (qRT-PCR)

To estimate the gene expression as well as miRNAs levels quantitative reverse transcriptase real-time PCR (qRT-PCR) was used. After 72 h, human ASCs were collected from biomaterials surface and polystyrene using trypsin solution (TrypLE™ Express, Thermo Fisher Scientific, Warszawa, Poland), centrifuged at 300× *g* for 4 min, and homogenized using TRI Reagent^®^ for RNA isolation. Total RNA was isolated using the phenol–chloroform method as previously described by Chomczynski and Sacchi [43]. The RNA was diluted in DEPC-treated water, then quantity and purity were estimated spectrophotometrically at 260 nm using NanoDrop 8000 (ThermoFisher Scientific, Waltham, MA, USA). Genomic DNA digestion and cDNA synthesis were performed using a PrimeScript kit (Takara, Clontech, Biokom, Janki, Poland). For the analysis of mRNA expression, cDNA was obtained from 1 µg of RNA using transcription Tetro cDNA Synthesis Kit (Bioline Reagents Ltd., London, UK). The synthesis of cDNA used for microRNA detection was performed using Mir-X miRNA First-Strand Synthesis Kit (Takara Clontech, Biokom, Janki, Poland) according to the protocol described previously [44,45]. The reactions of reverse transcription were performed on T100 Thermal Cycler (Bio-Rad Polska Sp. z.o.o., Warszawa, Poland). The qPCR reactions were carried out in a total volume of 20 μL reaction mix (SensiFast SYBR and Fluorescein Kit; Bioline Reagents Ltd., London, UK). The following transcripts were assessed: mitofusin-1 (*Mnf1*), mitochondrial fission 1 protein (*Fis1*), Beclin-1 (*Becn1*), Parkin (*Prkn*), tumor protein P53 (*p53*), cyclin dependent kinase inhibitor 1A (p21), survivin, B-cell lymphoma 2 gene (*Bcl-2*), and Bcl-2-associated X protein (*Bax*). The sequences of the primers used for amplification were described in detail in our previous work [43,44]. The sequences of primers used for microRNA (miR) detection are listed in Table 1. The specific oligonucleotide primer pairs were used at a concentration equal to 500 nM. The amplification efficiency of selected genes were normalized using the housekeeping genes—for mRNA, it was GAPDH (glyceraldehyde 3-phosphate dehydrogenase), and for miRNA, U6snRNA (primers supplied with the kit). The target genes’ relative quantification were evaluated using the comparative Ct method. qPCR was performed using CFX Connect Real-Time PCR Detection System (Bio-Rad Polska Sp. z.o.o., Warszawa, Poland).

### 2.7. Statistical Analysis

All experiments were triplicated. Data of porosity and swellability of sponges were analyzed by one-way factor analysis of variance (ANOVA), with a significance level defined at *p* ≤ 0.05 using Statistica 9. The analysis of data obtained in biological assays were conducted with STATISTICA 10.0 software (StatSoft, Inc., Statistica for Windows, Tulsa, OK, USA). The normality of the population data was determined using Shapiro–Wilk test, while equality of variances was assessed by Levene’s test. Differences between groups were determined using one- or two-way analysis of variance (ANOVA). Differences with a probability of *p* < 0.05 were considered as significant.

## 3. Results and Discussion

### 3.1. Structural Analysis of Nanocomposites

**Figure 2:**
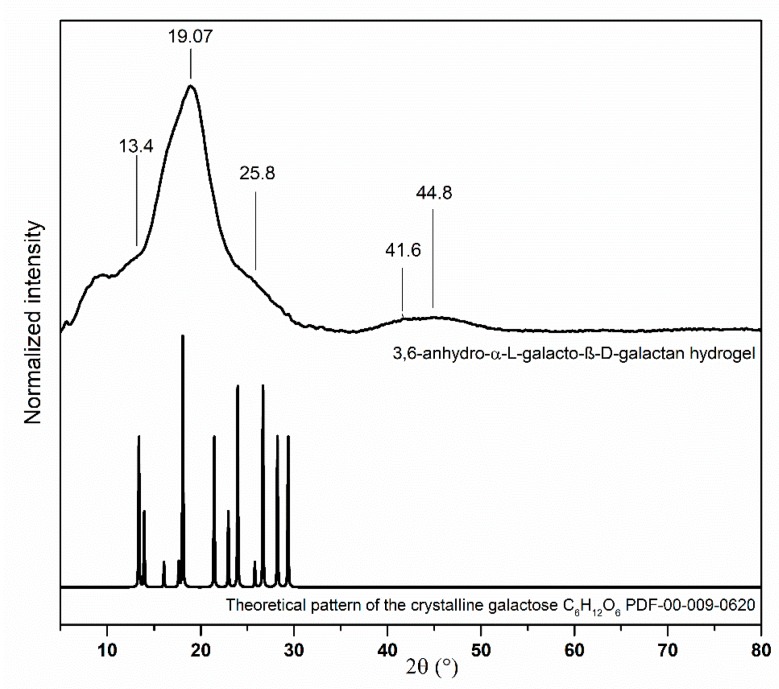
XRD pattern of galactose hydrogel.

The structural characterization of the samples was done by XRPD in a 2θ wide range. The XRPD pattern of pure 3,6-anhydro-α-l-galacto-β-d-galactan is presented in the Figure 2. All Bragg’s reflexes observing in the X-ray pattern correspond with the reference standard of the crystalline galactose (C_6_H_12_O_6_, PDF-00-009-0620). There are two main peaks at 8.32° and 19.07°. The broader one at 8.32° indicates an amorphous structure of hydrogel [46]. Intensity levels of two characteristic peaks for agar—polysaccharide composed of 3,6-anhydro-α-l-galacto-β-d-galactan—at 13.4° and 19.07° give information about the degree of crystallinity. The high intensity peak at 19.07° and only inflection point at 13.4 suggest the presence of a high content of amorphous phase [47]. 

**Figure 3:**
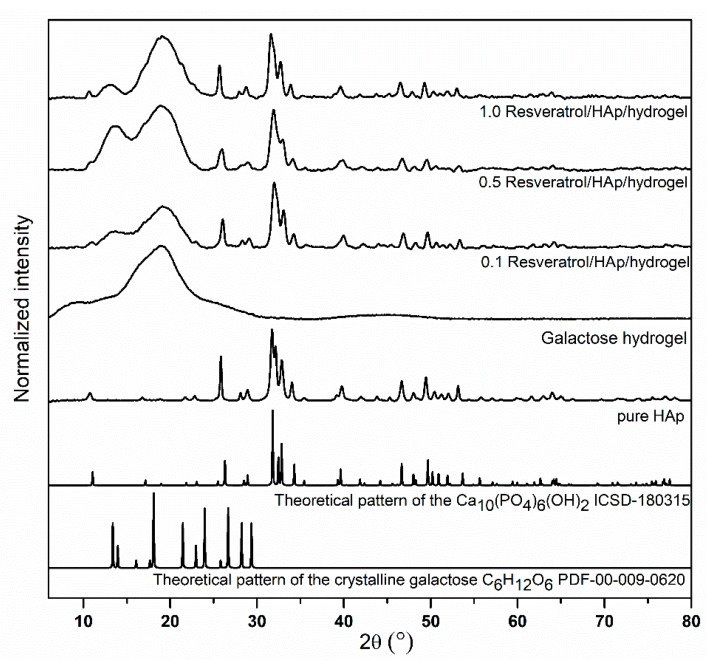
XRD spectra of hydroxyapatite, 3,6-anhydro-α-L-galacto-β-D-galactan and galactose hydrogels filled with resveratrol, at concentrations of 0.1, 0.5, and 1.0 mM, and at 1 mM concentration of hydroxyapatite.

X-ray powder diffraction of pure nanohydroxyapatite and samples filled with hydroxyapatite and resveratrol are presented in the Figure 3. As can be seen, the pure hexagonal phase corresponding to the standard reference of the Ca_10_(PO_4_)_6_(OH)_2_ (ICSD–180315) has been maintained, even after closing the nHAp in hydrogels loaded with resveratrol. The main peaks belonged to hydroxyapatite are located at 2θ, equal to 26.3°, 29.0°, 31.8°, 32.8°, 39.6°, 46.7°, 49.6°, and 53.4°, whereas the resveratrol (RES) peaks have been partly covered by the 3,6-anhydro-α-l-galacto-β-d-galactan peaks, being at 19.07°. Low concentrations of RES loaded in the 3,6-anhydro-α-l-galacto-β-d-galactan matrix lead to the lack of observation of peaks from drugs in that range. With more accurate analysis, it is possible to observe delicate inflection points and irregularities of the peak line at 22.6° and 23.3°, due to RES [48]. Similar results have been shown for other characteristic peaks belonging to RES. Peaks at 28.8° and 35.5° have been detected on XRD spectra, however, their intensity is low-lying. 

### 3.2. Spectroscopic Properties

**Figure 4:**
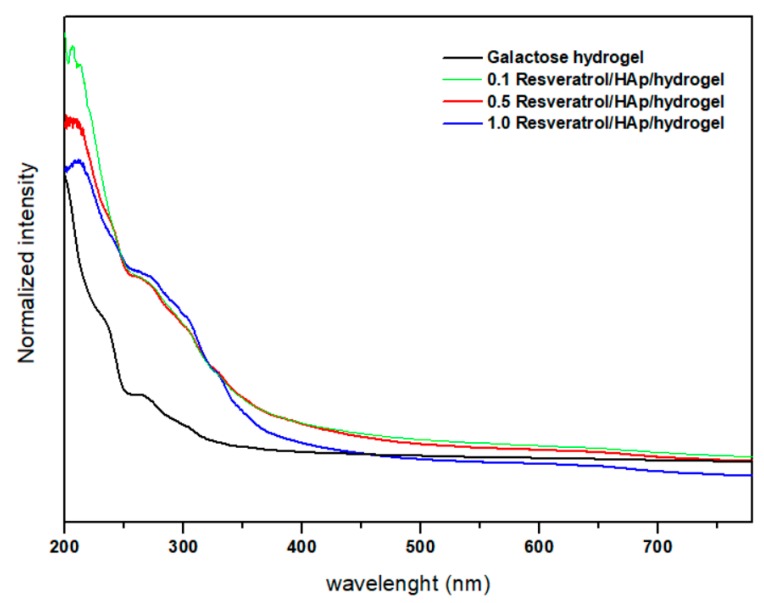
UV–Vis spectra of 3,6-anhydro-α-l-galacto-β-d-galactan hydrogel sample and samples filled with hydroxyapatite and resveratrol.

The absorption spectra (in the range of 200–800 nm) of the pure 3,6-anhydro-α-l-galacto-β-d-galactan, and filled with hydroxyapatite and resveratrol are presented in the Figure 4. UV–Vis spectra of the pure 3,6-anhydro-α-l-galacto-β-d-galactan are characteristic for the carbohydrate without absorption peaks in the UV–Vis range [49]. Since the 3,6-anhydro-α-l-galacto-β-d-galactan hydrogel matrix has a similar chemical structure as agar–agar polysaccharide, the sample of pure 3,6-anhydro-α-l-galacto-β-d-galactan does not absorb in the measured range [47], whereas the 0.1–1.0 Resveratrol/nHAp/hydrogel nanocomposites show an absorption peak at 300 nm, attributed to hydroxyapatite. Since a low concentration of resveratrol was used in each of the samples, characteristic peaks of resveratrol are not observed. 

**Figure 5:**
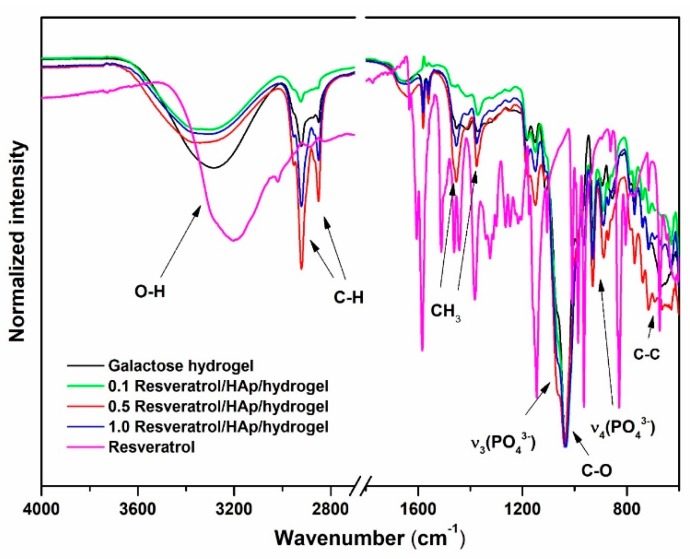
IR spectra of resveratrol, galactose hydrogel, 0.1 resveratrol/nHAp/hydrogel, 0.5 resveratrol/nHAp/hydrogel, and 1.0 resveratrol/nHAp/hydrogel.

To provide deeper insight regarding grafting of the resveratrol onto the surface of Ca_10_(PO_4_)_6_(OH)_2_ nanoparticles, as well as its influence on nanocomposition, solid state attenuated total reflectance infrared (ATR-IR) measurements were conducted. All of the ATR-IR spectra (see Figure 5) showed characteristic peaks in the range of 3351–3280 cm^−1^, attributed to O–H bands. Addition of hydroxyapatite and resveratrol shifted peaks by O–H bands to a higher frequency; that assigned in pure 3,6-anhydro-α-l-galacto-β-d-galactan hydrogel is located at 3284 cm^−1^, and from filled samples, at 3336 cm^−1^. The shifts for other peaks are not significant. The comparison of ATR-IR spectra of resveratrol and the nanocomposition showed that there is a major change in the fingerprint region, i.e., 830 to 1202 cm^−1^, as well as 3200 cm^−1^. First of all, there are intense peaks due to βOH and γCH, typical in the spectrum of resveratrol, which have disappeared into the nanocomposite. Secondly, there is broad peak due to O–H stretching, typical in the spectrum of galactose hydrogel, which is shifted to 3334 cm^−1^ in the complex, indicating there is interaction of OH groups of hydrogel with resveratrol as well. The peaks at 1640 cm^−1^ are assigned to C–O bands. The byproduct of the polymerization reaction is 3,6-anhydro-α-l-galacto-β-d-galactan, with characteristic peaks at 1075 cm^−1^, 1039 cm^−1^, and 930 cm^−1^, attributed to the C–O bands [47,50]. The ATR-IR spectra presented in the Figure 5 show the abovementioned peaks. The peak at 889 cm^−1^ demonstrates the C–H residual carbon stretching of 3,6-anhydro-α-l-galacto-β-d-galactan [49,51]. The modes around 1060 cm^−1^ are characteristic for the triply degenerated ν_3_ anti-symmetric stretching of the P–O groups belonging to nanohydroxyapatite.

Seeing that the ATR-IR spectra of 3,6-anhydro-α-l-galacto-β-d-galactan composite are similar to the spectra of pure 3,6-anhydro-α-l-galacto-β-d-galactan hydrogel, it could be assumed that there were no significant chemical bonds between hydroxyapatite, resveratrol, and hydrogel matrix.

### 3.3. Resveratrol Release from 3,6-Anhydro-α-l-galacto-β-d-galactan Hydrogel Filled with Resveratrol and Hydroxyapatite

**Figure 6:**
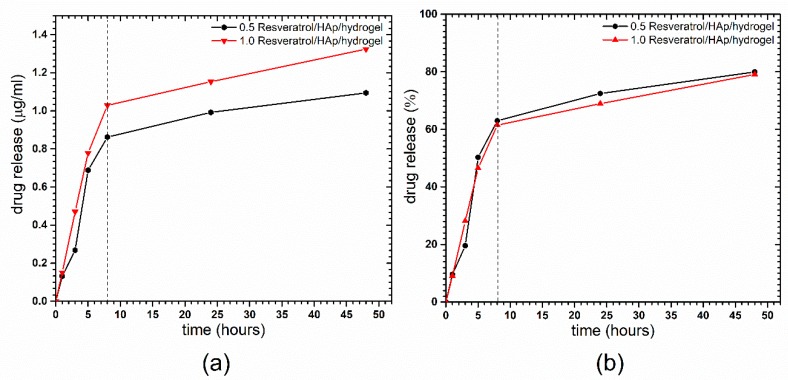
Time dependent the resveratrol release from the loaded nHAp/hydrogel presented as released mass (**a**) of the RES and its percentage (**b**).

The results of the time-dependent resveratrol (RES, *trans*-3,5,4′-trihydroxystilbene) release from the compound loaded on the nHAp/hydrogel are shown in the Figure 6, presented as released mass (a) of the RES and its percentage (b). At the first step, an increase of resveratrol concentration in buffer solution at pH 6.8 was observed after 8 h (approximately 60%). Afterwards, a slow release of the RES has been detected. As can be seen, RES release is not instantaneous, and the highest dose can be detected after 45 h. It has to be underlined that, after 45 h, saturation of the solution is achieved, meaning that the equilibrium has been reached. This is the standard test of compound release performed in a closed circuit, without fluid exchange, and it should be emphasized that the concentration of RES after 45 h could be constant over longer periods of time.

### 3.4. SEM Analysis of Nanocrystalline Hydroxyapatite Loaded with Resveratrol in Colloidal Suspension Structure

**Figure 7:**
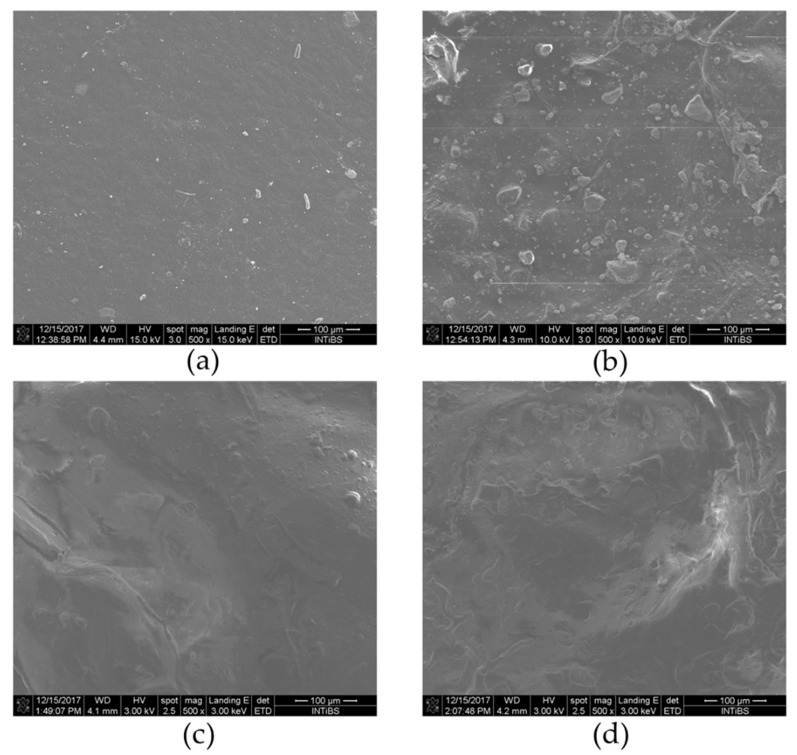
SEM of (**a**) galactose hydrogel, (**b**) 0.1 resveratrol/nHAp/hydrogel, (**c**) 0.5 resveratrol/nHAp/hydrogel, and (**d**) 1.0 resveratrol/nHAp/hydrogel. Magnification 500×.

The morphology of pure 3,6-anhydro-α-l-galacto-β-d-galactan hydrogel (a), and filled with resveratrol and hydroxyapatite (a–d) are presented in the Figure 7. The pure 3,6-anhydro-α-l-galacto-β-d-galactan hydrogel presented homogenous morphology, while the addition of nHAp results in the creation of rough morphology. A similar observation has been made by other authors using SEM [52,53]. Moreover, it was observed that increased amounts of RES lead to that creation of complex aggregates in micrographs as segments with round/spherical structures (c, d). The different morphology in obtained materials might be an important factor that correlates with scaffold cytocompatibility, and might positively correlate with cell proliferative and metabolic activity (see Section 3.5).

### 3.5. The Influence of Hydrogels Functionalized with nHAp and Resveratrol on hASC Morphology, Proliferation, and Metabolic Activity

**Figure 8:**
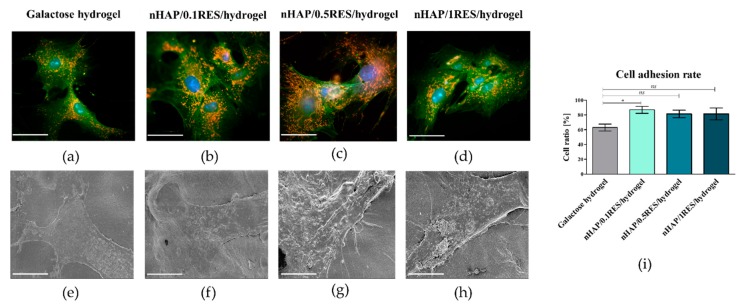
The images obtained using epifluorescence microscope (**a**–**d**) and SEM (**e**–**h**). The cultures of hASCs were cultured on galactose hydrogel (**a**,**e**), and biomaterials functionalized with 0.1 resveratrol (**b**,**f**), 0.5 resveratrol/nHAp/hydrogel (**c**,**g**), and 1.0 resveratrol/nHAp/hydrogel (**d**,**h**). The results of adhesion rate noted for hASCs cultured on tested scaffolds **(i)**. Comparative analysis of adhesion rates revealed that nHAp/hydrogel functionalized with 0.1 mM of resveratrol significantly (* *p* < 0.05) improves the adhesion of hASCs. Non-significant (*ns*) increase of adhesion rate was noted in cultures on nHAp/hydrogel with 0.5 and 1 mM of resveratrol. The EpiFM observations were carried out under 60-fold magnification. The scale bar is 40 μm. The following structures of cells were visualized: actin cytoskeleton (phalloidin—green), nucleus (DAPI—blue), mitochondria (MitoRed—orange). The SEM images were captured under 2500-fold magnification; scale bar is 4 μm.

The analysis revealed that functionalization of hydrogels with nHAp and resveratrol positively affected the growth pattern and metabolic activity of hASCs. The cells cultured with all obtained hydrogels maintained their proper fibroblast-like morphology, however, the functionalization of hydrogels with nHAp and resveratrol distinctly promoted cell–cell interactions and enhanced cell adhesion to the biomaterial (Figure 8a–h). Increased adhesion of hASCs onto functionalized hydrogels was observed, but the significant difference in cell adhesion rate was noted in cultures on 0.1 mM resveratrol/nHAp/hydrogel. Both nHAp as well as RES were noted as factors improving the adhesion and spreading of cells (Figure 8i).

**Figure 9:**
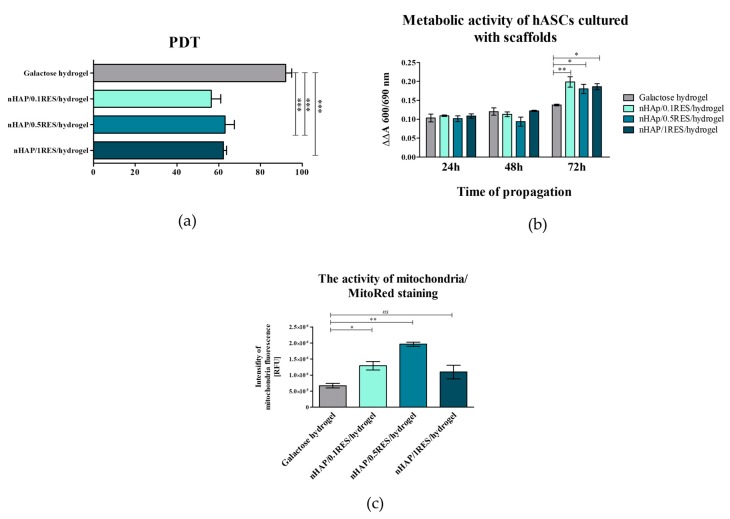
The influence of resveratrol/nHAp/hydrogels on hASC population doubling time (**a**), and metabolic (**b**) and mitochondrial activity (**c**). Statistically significant changes are indicated with asterisks, (*) *p* < 0.05, (**) *p* < 0.01, and (***) *p* < 0.001, while non-significant differences are marked as *ns*.

In this study, the modulatory effect of galactose hydrogel loaded with nHAp at different concentrations of resveratrol on the proliferation and metabolic activity of hASCs has been determined. It has been found that functionalization of galactose hydrogels with nHAp and RES influences hASC proliferation significantly, reducing the time needed for cell doubling. The mean population doubling time (PDT) noted for hASCs in cultures on galactose hydrogels was 92 (±6) h, while cultures functionalized with 0.1 mM resveratrol/nHAp or 0.5 mM resveratrol/nHAp and 1 mM resveratrol/nHAp had a population doubling time established at 56 (±9), 63 (±9), and 62 (±3) h, respectively (Figure 9a). The wealth of literature underscores the fact that functionalization of composites with nanosized or microsized nHAp have advantages in terms of their increased cytocompatibility and future biomedical applications as a carrier for progenitor cells. The bioactivity of scaffolds, related to their supporting role as matrices in promoting initial attachment and having pro-proliferative function, is obvious in the context of cell-based therapies [54]. For example, it was shown that polydimethylsiloxane (PDMS) matrix functionalized with nHAp simultaneously promoted attachment and proliferation of a human osteoblast-like MG-63 cell line. Additionally, nHAp films improve proliferation of bone marrow-derived mesenchymal stem cells (BMSCs). The doubling time of BMSCs in cultures on nHAp films was significantly shorter than that on films containing larger particles of HAp (i.e., conventional HAp) [55]. Resveratrol was also indicated as an agent positively affecting proliferation and osteoblastic maturation of human BMSCs cultures. Peltz et al. [56] emphasized the fact that the effect of resveratrol on human adipose-derived mesenchymal stromal cells is dosage dependent, and is a result of its combinatorial effect on cell senescence rate, cell doubling time, and cell proliferation rate. In that study, it was shown that hASC self-renewal potential was slightly enhanced by resveratrol at 0.1 mM, unchanged at 1 mM, but significantly inhibited at 5 or 10 mM, regardless of the pretreatment duration. In our study, the dose-dependent effect was not clear. Indeed, the hydrogels functionalized with 0.1 mM resveratrol/nHAp exerted the most beneficial effect in terms of cells attachment and metabolic activity, while the effect of hydrogels loaded with 0.5 and 1 mM resveratrol nHAp was comparable in this manner. These results also showed that hydrogels functionalized with nHAp and resveratrol enhanced the hASC adhesion rate (Figure 8i). Moreover, the analysis of metabolic activity of hASCs in cultures with galactose hydrogels showed that functionalization of biomaterials with resveratrol/nHAp did not affect cell activity during the first 48 h. The significant increase in metabolic activity of in hASCs resulted from hydrogels functionalization was noted after 72 h of propagation (Figure 9b). Increased metabolic activity of hASCs cultured on galactose hydrogels loaded with resveratrol/nHAp correlated with increased activity of mitochondria, which was observed in cultures stained with MitoRed (Figure 8a–d). The analysis of staining intensity revealed that the number of active mitochondria in hASCs cultured on 0.1 mM resveratrol/nHAp and 0.5 mM resveratrol/nHAp hydrogels significantly increased (Figure 9c). Taking into account the results of hASCs metabolic activity in cultures with tested biomaterials, we were interested in their role on modulation of mitochondrial activity.

### 3.6. The Influence of Hydrogels Functionalized with nHAp and Resveratrol on hASCs Reactive Oxygen Species and Mitochondria Potential

**Figure 10:**
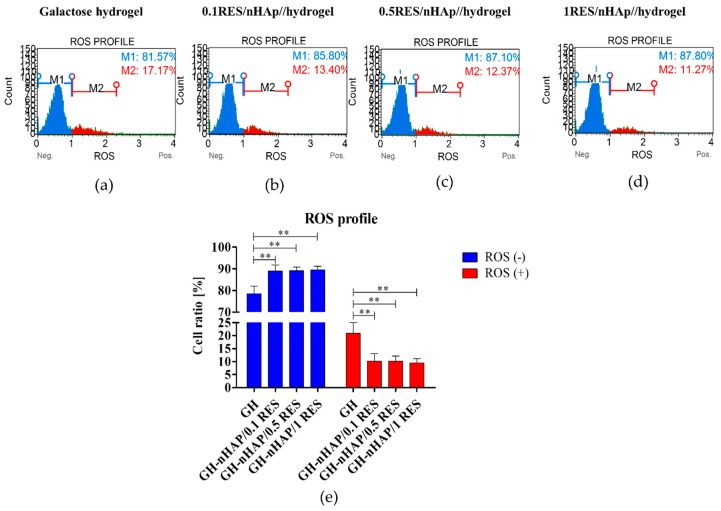
The results of analysis of reactive oxygen species (ROS) accumulation in hASC cultured on tested hydrogels. The representative histograms (**a**–**d**) showing the distribution of cells into two populations—cells not showing activity or ROS, and cells accumulating ROS. M1 refers to the ROS-negative population of cells and M2 indicates ROS-positive cells. The comparative analysis was performed (**e**) and statistically significant changes are indicated with asterisks (**) *p* < 0.01.

Interestingly, both enhanced metabolic activity and viability were positively correlated with significantly reduced reactive oxygen species level, i.e., ROS, which suggests effective antioxidative activity of 0.1 mM resveratrol/nHAp. Oxidative stress is recognized as a major factor contributing to the development of age-related diseases. The excessive accumulation of ROS, combined with reduced level of superoxidase dismutase (SOD), impairs, in hMSCs, their osteogenic differentiation potential, which finally limits their clinical application. In this study, it was shown that 0.1 mM resveratrol/nHAp reduces the number of ROS^positive^ hASCs, and simultaneously increased the number of ROS^negative^ hASCs (Figure 10). It was shown that ROS inhibit MSC proliferation, increase senescence, enhance adipogenic but reduce osteogenic differentiation, and inhibit MSC immunomodulation [57]. Moreover, in our previous research, it has been shown that polyurethane–polylactide (PU/PLLA), based on material filled with RES, decreases ROS levels in cultures of progenitor cells, and thus reduced senescence and oxidative stress in hASCs [23]. 

**Figure 11:**
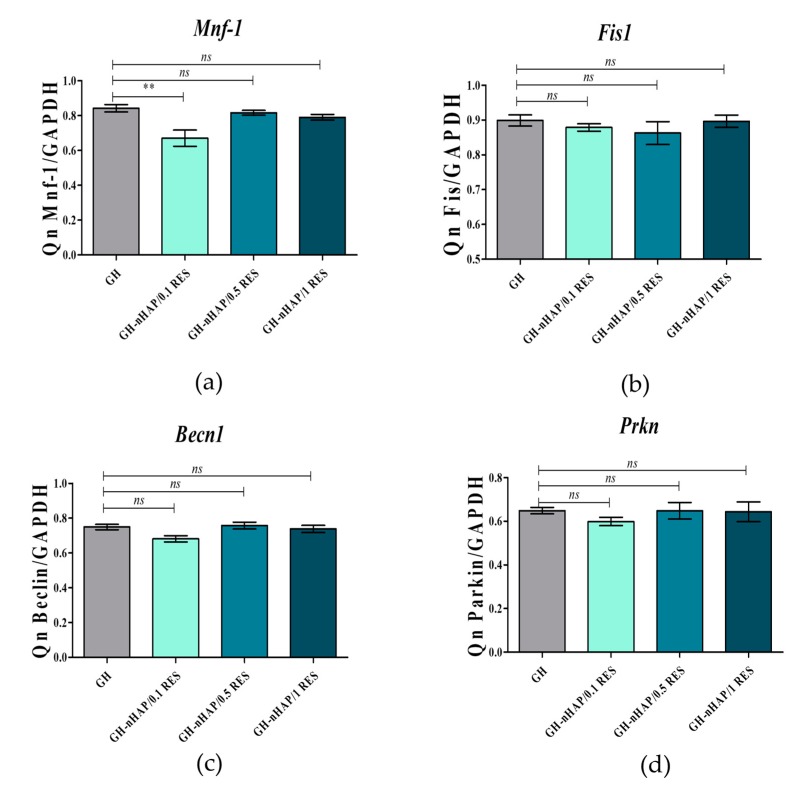
The mitochondrial fusion gene expression of mitochondrial fusion (**a**), mitochondrial fission 1 protein (**b**), Beclin-1 (**c**), and Parkin (**d**) in hASCs. Statistically significant changes are indicated with asterisks; (**) *p* < 0.01, while non-significant differences are marked as *ns*.

The reduced accumulation of ROS was positively correlated with increased mitochondrial potential in hASCs cultivated onto 1 mM resveratrol/nHAp (Figure 9d). Moreover, the observed effect was accompanied by significantly reduced mitofusin-1 (MNF-1) expression according to mRNA levels; mitofusin is an essential transmembrane GTPase that mediates mitochondrial fusion (Figure 11a). It was also analyzed whether other genes, such as mitochondrial fission 1 protein (*Fis1*), Beclin-1 (*Becn1*), and Parkin (*Prkn*) are involved in the cell response to hydrogels functionalized with nHAp and resveratrol, but no statistically significant changes were observed (Figure 11b–d). These results indicate proper mitochondrial metabolism. *Beclin 1* and *Parkin* are genes having pro-autophagic activity [58,59]. Similarly, *Fis1* is a gene that induces mitochondrial fragmentation and enhances the formation of autophagosomes [60]. The constant (constitutive) expression of mRNA for those genes, noted in cultures with hydrogel scaffolds, indicates cellular homeostasis and maintenance of mitochondrial integrity. 

Mitochondrial dynamics, that are regulated by fusion and fission, have been proposed to form a quality control mechanism that allows removal of damaged mitochondria from the cell, and thus protects against senescence and aging [61]. Although no correlation between autophagy and mitochondrial fusion has been demonstrated in this study, the reduced mitochondrial fusion combined with reduced ROS^positive^ cells cultured onto 0.1 mM resveratrol/nHAp shed a promising light for that material in the context of its antioxidative effect. Interestingly, there was no correlation between mitochondrial fission between the investigated groups, which indicated on inhibitory effect of RES on mitochondrial fission. Obtained results positively correlate with observed increased viability and metabolic activity of hASCs cultured onto 0.1 mM resveratrol/nHAp, since inhibition of mitochondrial fission has been showed to increase cell viability, improve cellular alkaline phosphatase (ALP) activity and mineralization, and restore mitochondrial function [61]. 

**Figure 12:**
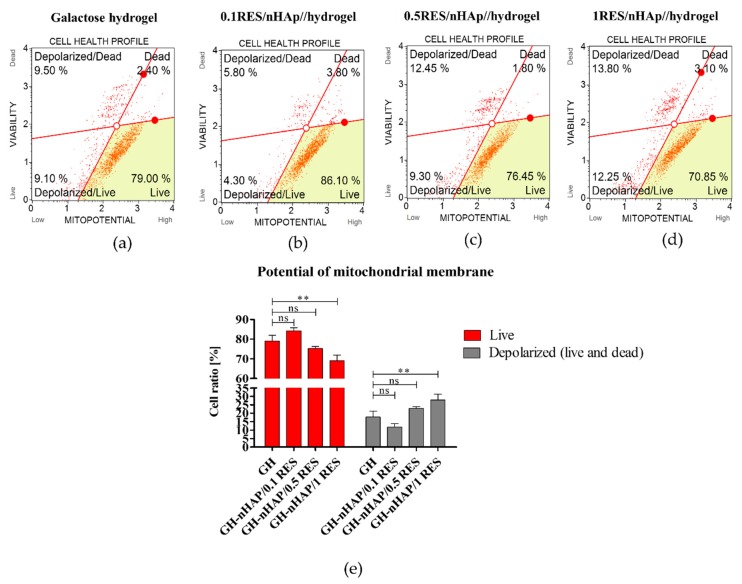
The potential of mitochondrial membrane in hASCs combined with resveratrol/nHAp/hydrogels. Representative images showing distribution of cells based on the mitochondrial membrane potential in cultures with native hydrogel scaffold (**a**), and hydrogel functionalized with nHAp and 0.1 mM of resveratrol (**b**), 0.5 mM of resveratrol (**c**), and 1 mM of resveratrol (**d**). Changes were analyzed for statistical significance (**e**), which is indicated with asterisks; (**) *p* < 0.01, while non-significant differences are marked as *ns*.

The present study confirmed that nanocrystalline calcium hydroxyapatite (Ca_10_(PO_4_)_6_(OH)_2_—nHAp) doped with resveratrol in a dose-dependent manner improves viability and metabolic activity in human adipose-derived mesenchymal stem cells (hASCs) through improved mitochondrial potential. The grater viability and metabolic activity of hASCs cultured onto the lowest dosage of resveratrol, e.g., 0.1 mM resveratrol/nHAp correlates with the highest percentage of living cells among other groups. Furthermore, the improved viability of hASCs cultured onto 0.1 mM resveratrol/nHAp materials was accompanied by the lowest percentage of early and late apoptosis of cells (Figure 12). Moreover, the greatest metabolic activity that was observed was positively correlated with the most expanded morphotype of hASCs cultured onto 0.1 mM resveratrol/nHAp.

### 3.7. Apoptosis Analysis of hASCs with Resveratrol/nHAp/Hydrogels

**Figure 13:**
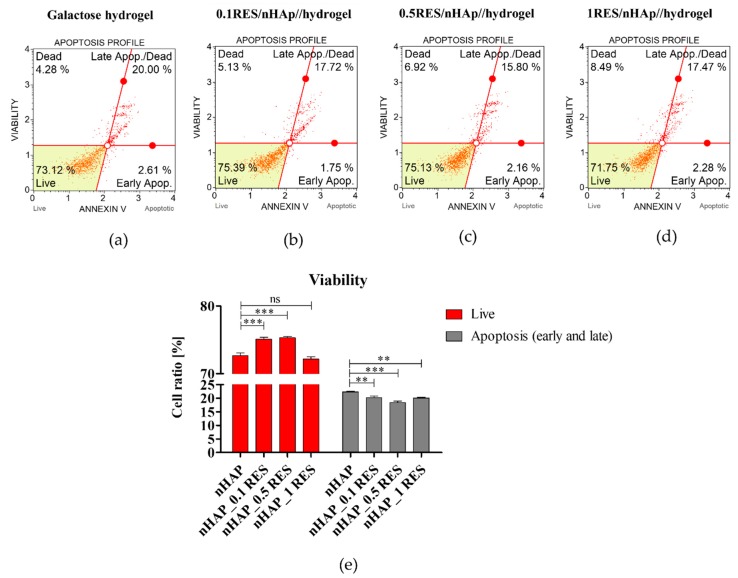
The influence of nHAp functionalized with resveratrol on cells viability. Representative images showing distribution of cells based on the viability noted in cultures with native hydrogel scaffold (**a**), and hydrogel functionalized with nHAp and 0.1 mM of resveratrol (**b**), 0.5 mM of resveratrol (**c**), and 1 mM of resveratrol (**d**). The statistical analysis of obtained results was performed (**e**), and significant changes are indicated with asterisks, (**) *p* < 0.01 and (***) *p* < 0.001, while non-significant differences are marked as *ns*.

To determine whether the used biomaterial influences on viability of hASC and induction of apoptosis, we measured the percentage of apoptotic cells using an annexin V staining protocol. The obtained results show that nHAp functionalized with resveratrol increased the cell viability, simultaneously reducing the number of apoptotic cells. We noticed that these effects were observed after hASCs’ exposition to nanohydroxyapatite functionalized with resveratrol using three different dosages (Figure 13). 

**Figure 14:**
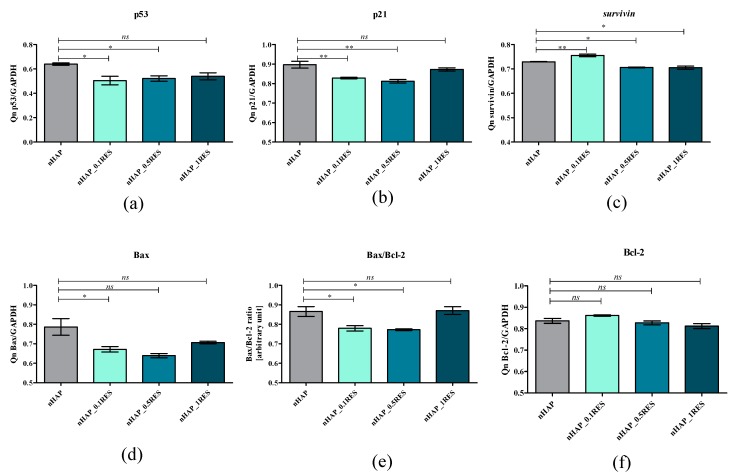
Relative expression of genes involved in the regulation of apoptosis *p53* (**a**), *p21* (**b**), *survivin* (**c**), *Bax* (**d**), and *Bcl-* (**e**) normalized to expression of GAPDH. The expression ratio of *Bax* and *Bcl-2* in human ASCs (**f**). Statistically significant changes are indicated with asterisks, (*) *p* < 0.05 and (**) *p* < 0.01, while non-significant differences are marked as *ns*.

To find out which molecular factors can be responsible for viability enhancement and depletion apoptosis, we analyzed the expression of the following genes involved in the process of apoptosis: *p21*, *p53*, *survivin*, *Bax*, and *Bcl-2*. In this study, 0.1 resveratrol/nHAp/hydrogel significantly reduced activity of *p21*, *p53*, and *Bax* transcripts, which was accompanied by an increased expression of *Bcl-2* (Figure 14a,b,d,f). The *Bcl-2* gene has generally anti-apoptotic qualities, while *Bax* is pro-apoptotic. Both genes play an important role in the regulation of the apoptosis in various types of cells [62]. The proportion of *Bcl-2* or *Bax* can determine whether a cell is protected, or otherwise, from apoptosis [63]. We observed that the level of pro-apototic gene *Bcl-2* was higher compared to *Bax*. Observed phenomenon was positively accompanied by an observed greater expression of *survivin* (Figure 14c), which is recognized as a master regulator of MSC activity and functionality. *Survivin* was showed to be an inhibitor-of-apoptosis protein (IAP) family member and plays an important role in the regulation of apoptosis, cell division, and cell cycle control—all crucial for the regenerative ability of MSCs [64]. Obtained data correlate with previous findings, which demonstrated that RES dose-dependently reduces apoptosis and promotes cell self-renewal by inhibition of cellular senescence [56].

### 3.8. The Role of microRNAs after hASCs’ Resveratrol/NHAp/Hydrogels Treatment

**Figure 15:**
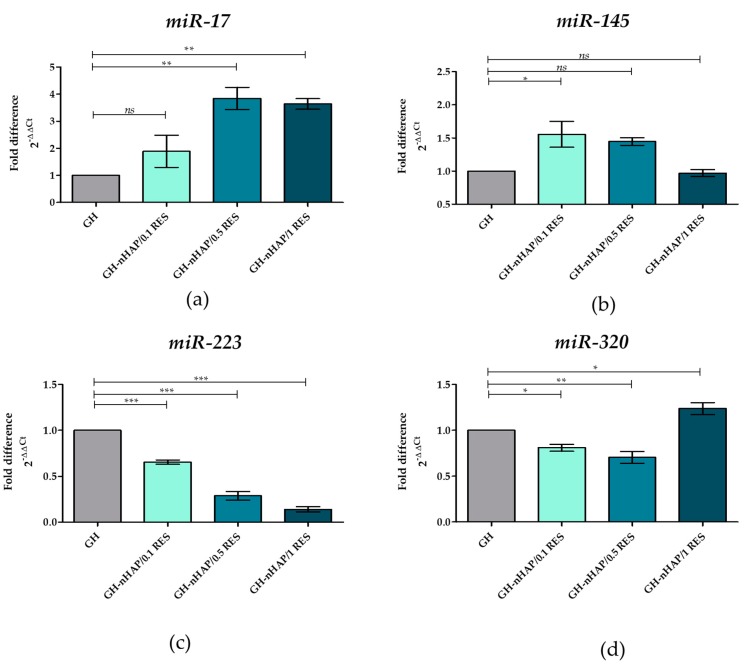
Relative expression of microRNAs after hASCs’ resveratrol/nHAp/hydrogels incubation. Analysis included determination of *miR-17* (**a**), *miR-145* (**b**), *miR-223* (**c**), and *miR-320* (**d**). Statistically significant changes are indicated with asterisks, (*) *p* < 0.05, (**) *p* < 0.01 and (***) *p* < 0.001 while non-significant differences are marked as *ns*.

In this study, the positive correlation between reduced apoptosis and increased expression of miR-145 and miR-17 was demonstrated. The both miR-145 and miR-17 have been shown to be crucial regulators of osteogenic differentiation, which target BMP2 and BMPR2 through Smad1/7 [65]. It is also known that p53 upregulates miR-145 expression. This correlation strongly influenced the improvement of HSC self-renewal [66]. In our study, we observed that the mRNA level of p53 was downregulated (Figure 14a) while, simultaneously, the level of miR-145 was upregulated (Figure 15b). Therefore, the observed increase in hASC viability may be due to the effect of p53 on miR-145 level. In turn, the secondarily increased miR-17 level could also be responsible for increasing cell viability. Other authors have shown that resveratrol upregulates miR-17 in human keratinocytes cells (HaCaT) after injury. The underlying mechanism of cytoprotection of RSE was explored, and miR-17 expression was found to be upregulated after HaCaT cells were incubated with RSE. Inhibiting miR-17 expression impaired the growth-promoting effect and inflammation-inhibitory effect of RSE. They conclude that RSE protected HaCaT cells by upregulation of miR-17 [67]. Interestingly, we observed that level of miR-17 increased under the resveratrol/nHAp/hydrogels influence (Figure 15a). This may be explained by hASC viability increasing after resveratrol/nHAp/hydrogels stimulation due to increased amounts of miR-17.

We also observed a positive correlation between reduced apoptosis and decreased expression of *miR-223* and *miR-320*. The reason for the reduction in the percentage of human apoptotic ASC may be a decrease in the level of *miR-223* and *miR-320* molecules. Overexpression of *miR-223* in Hep3B cells significantly suppressed cell proliferation and promoted apoptosis [68]. A similar effect was observed in the studies on AML cells, whereby overexpression of *miR-223* reduced cell proliferation and promoted cell apoptosis, additionally suggesting a suppressive role for *miR-223* [69]. In our study, the application of resveratrol/nHAp/hydrogels resulted in a significant reduction in the level of *miR-223* in a dose-dependent manner (Figure 15c), and depleted the apoptosis of hASCs. It is known that *miR-320* inhibition decreased the rate of apoptosis in vivo and in vitro by increasing Bcl-2 expression and decreasing Bax. *miR-320* inhibition suppresses the apoptotic effect by affecting the levels of anti-apoptotic Bcl-2 and pro-apoptotic Bax [70]. Other studies have demonstrated that overexpression of *miR-320* reduces the protein level of anti-apoptotic Bcl-2 [71]. We observed a similar relationship in our research, with the decreasing of *miR-320* (Figure 15d) and the slightly increased level of *Bcl-2* (Figure 14f), while the *Bax* (Figure 14d) level was significantly reduced. Summarizing, the increase in the level of *miR-17* and *miR-145* molecules and the simultaneous reduction in the amount of *miR-223* and *miR-320* in the presence of resveratrol/nHAp/hydrogels protect hASCs against apoptosis. 

## 4. Conclusions

The obtained results indicate that nanocrystalline hydroxyapatite loaded with resveratrol in colloidal suspension improves viability, metabolic activity, and mitochondrial potential in human adipose-derived mesenchymal stromal stem cells (hASCs). Obtained biomaterials might be helpful in the field of elderly patient bone regeneration and may find application in broadly defined tissue engineering. Further animal studies are required to implement the obtained data to clinical science. 

## Figures and Tables

**Table 1:** Sequences of primers used for detection of microRNA.

miRNA	Specific Primer Sequence	Accession Number
hsa-miR-17-5p	CAAAGTGCTTACAGTGCAGGTAG	MIMAT0000070
hsa-miR-145-5p	GTCCAGTTTTCCCAGGAATCCCT	MIMAT0000437
hsa-miR-223-3p	TGTCAGTTTGTCAAATACCCCA	MIMAT0000280
hsa-miR-320a-3p	AAAAGCTGGGTTGAGAGGGCGA	MIMAT0000510
